# Herpes zoster-associated cutaneous vasculitis following intra-articular triamcinolone acetonide injection in an immunocompetent patient

**DOI:** 10.1016/j.jdcr.2026.07.027

**Published:** 2026-07-23

**Authors:** Dhiren R. Rajagopal, Dipti Anand, Melissa D. Babcock

**Affiliations:** a Georgia Institute of Technology, Atlanta, Georgia; b Babcock Dermatology, Atlanta, Georgia; c SkinPath Solutions, Smyrna, Georgia

**Keywords:** intra-articular steroids, leukocytoclastic vasculitis, varicella-zoster virus

## Introduction

Herpes zoster (HZ) results from reactivation of latent varicella zoster virus (VZV) within dorsal root ganglia and is associated with well-established risk factors, including increasing age and impaired cell-mediated immunity.^1^ Although systemic corticosteroids impair T-cell–mediated antiviral responses and increase the risk of HZ,^2^^,^^3^ the role of localized corticosteroid administration, particularly intra-articular injection, remains less well defined and appears to be uncommon.^4^^,^^5^

Rarely, herpes zoster may rarely manifest as cutaneous small-vessel vasculitis, which is most frequently reported in immunocompromised individuals.^6^^,^^7^ Fewer than a dozen cases of HZ-associated leukocytoclastic vasculitis have been described in the literature, and reports involving immunocompetent patients are exceedingly limited. To our knowledge, no prior reports have described the coexistence of HZ-associated cutaneous vasculitis following intra-articular corticosteroid injection in an immunocompetent patient.

Here, we present such a case and discuss potential mechanisms linking localized corticosteroid administration, viral reactivation, and vascular inflammation.

## Case report

A 70-year-old man with a history of right knee osteoarthritis presented with a painful eruption on the right lower extremity that began 5 days after intra-articular triamcinolone acetonide injection consisting of 2 mL of a 40 mg/mL solution (total dose 80 mg). He had no history of immunosuppression, malignancy, or chronic systemic corticosteroid use.

The eruption initially consisted of grouped vesicles that progressed over several days to hemorrhagic, crusted erosions in a dermatomal distribution along the anterior aspect of the right lower leg. The patient reported localized pain but denied fever, chills, or systemic symptoms. On physical examination, grouped hemorrhagic crusted erosions and vesicles were present on the anterior right lower leg in a dermatomal pattern (Fig 1).

**Fig 1:**
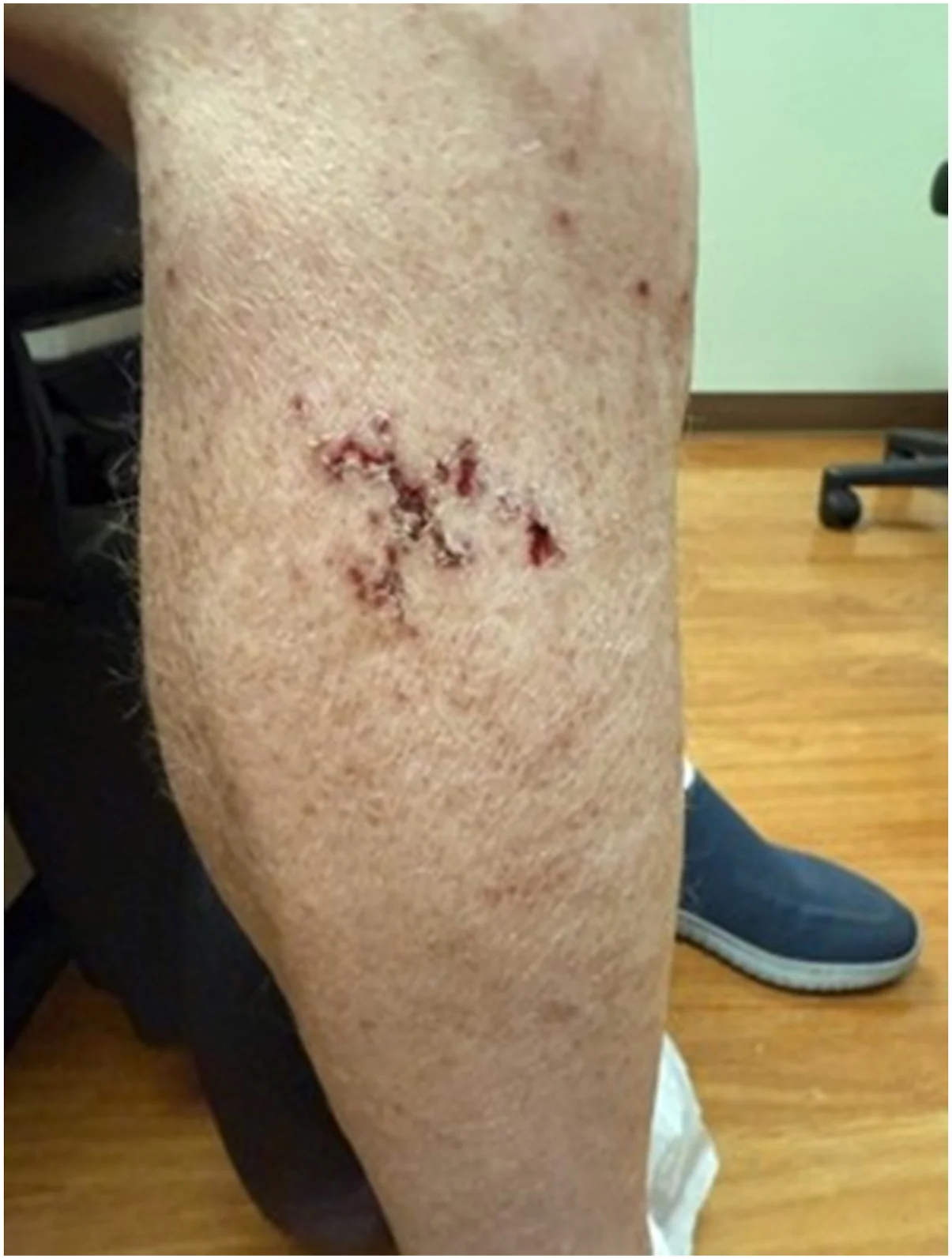
Grouped hemorrhagic crusted erosions on the anterior surface of the right lower extremity.

A punch biopsy of a representative lesion was performed. Histopathologic examination demonstrated ulceration with epidermal necrosis and an associated superficial and deep dermal perivascular lymphocytic inflammatory infiltrate (Fig 2, *A*). Higher magnification revealed degenerated epithelial cells with viropathic changes, including multinucleation and chromatin margination (Fig 2, *B*). Immunohistochemical staining for VZV was strongly positive in lesional epithelial cells (Fig 2, *C*), supporting the diagnosis of herpes zoster. The diagnosis of leukocytoclastic vasculitis (LCV) was supported by the presence of fibrinoid vascular necrosis with leukocytoclasia and hemorrhage (Fig 3).

**Fig 2:**
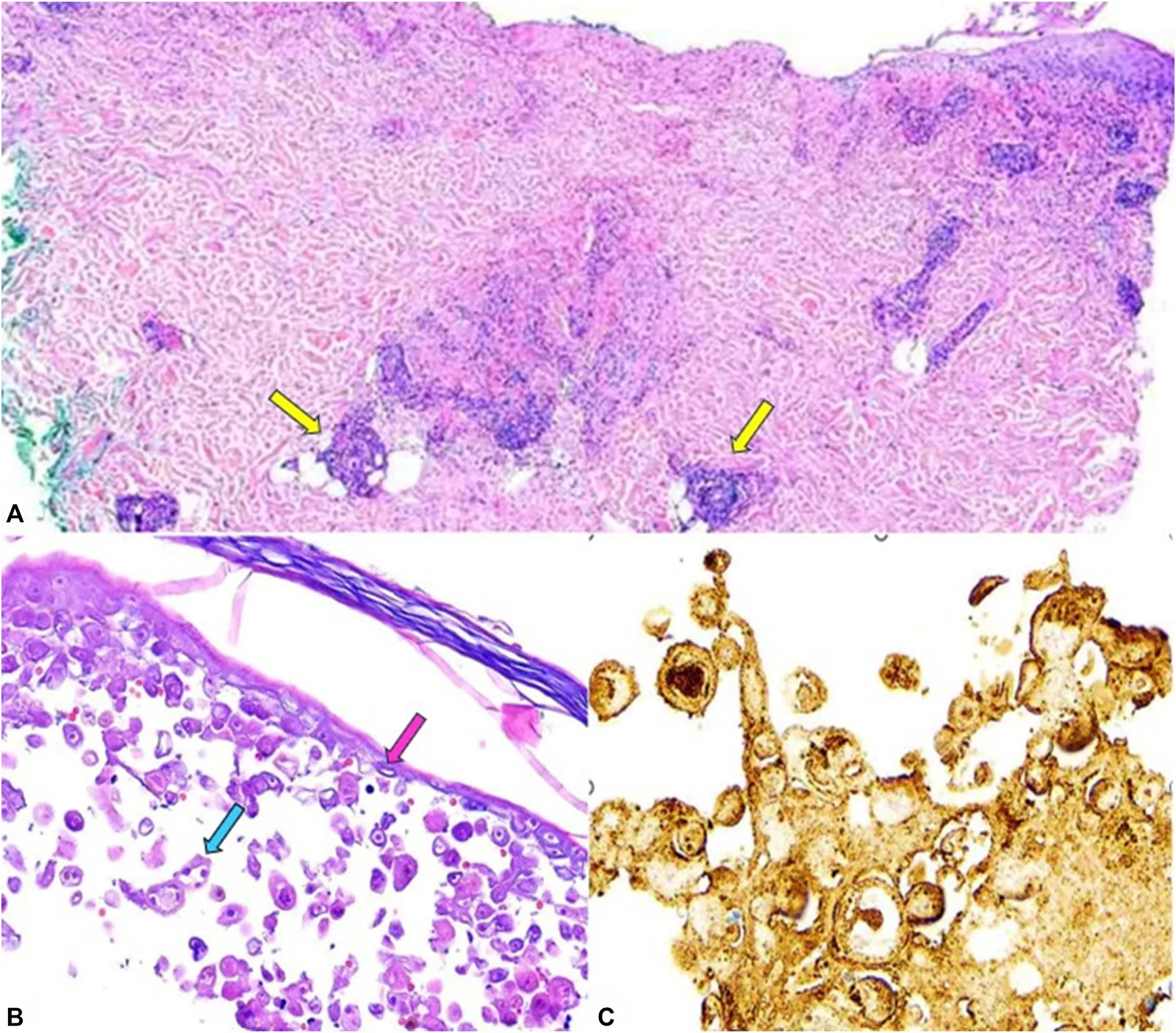
**A,** Low-power view (2×) demonstrating superficial and mid-dermal perivascular inflammatory infiltrates (*yellow arrows*). Hematoxylin and eosin stain. **B,** High-power view (30×) showing viral cytopathic changes, including multinucleation (*light blue arrow*) and chromatin margination (*magenta arrow*). Hematoxylin and eosin stain. **C,** Varicella-zoster virus immunostain highlighting viral antigen within lesional keratinocytes. 3,39-diaminobenzidine chromogen with hematoxylin counterstain.

**Fig 3:**
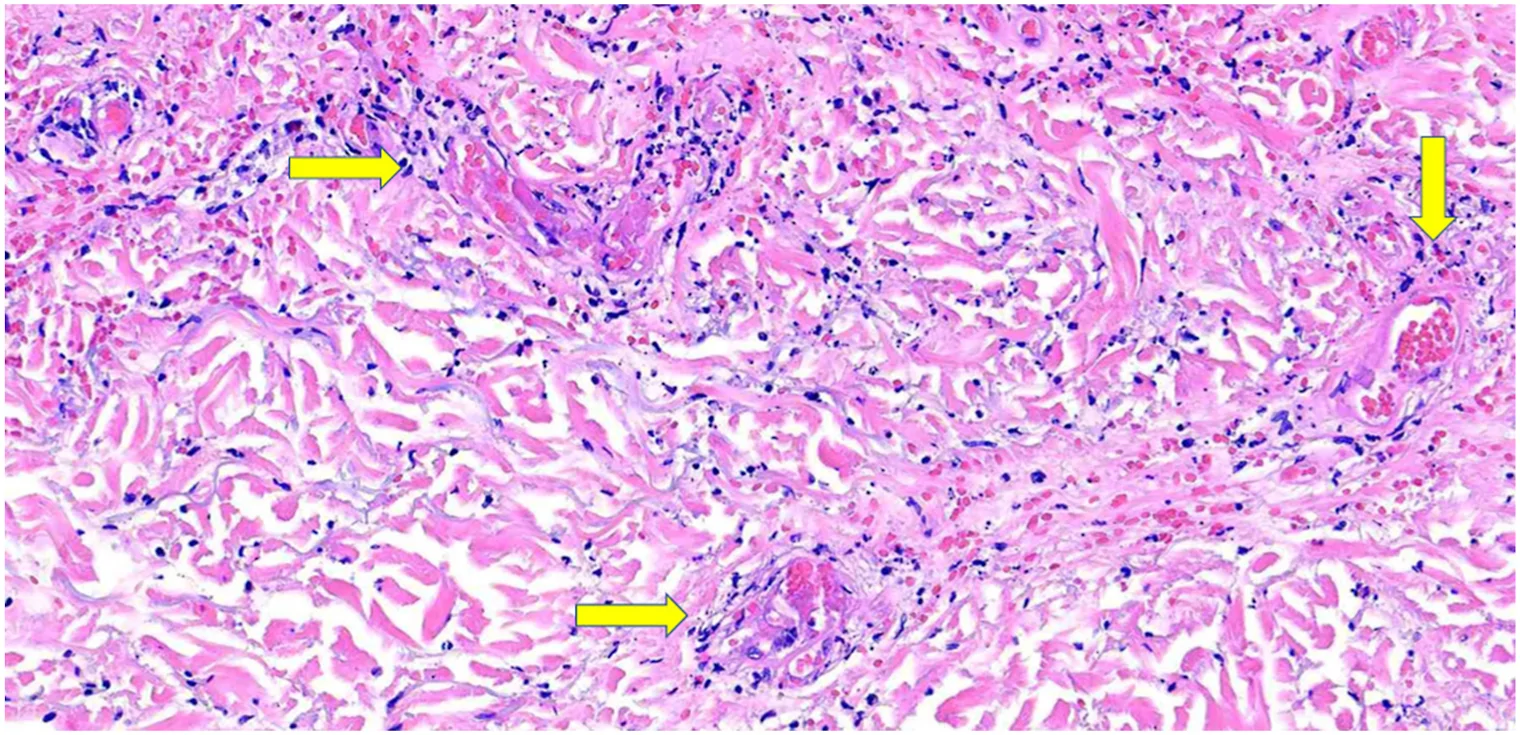
High-power view (30×) showing fibrinoid vascular necrosis with leukocytoclasia and hemorrhage. Hematoxylin and eosin stain (*yellow arrows*).

Based on clinical, histopathologic, and immunohistochemical findings, a diagnosis of herpes zoster with associated cutaneous LCV was established. The patient was treated with a 7-day course of valacyclovir and triamcinolone 0.1% topical cream, and the eruption resolved completely within 2 weeks, without recurrence or postherpetic neuralgia at 3 months of follow-up.

## Discussion

Herpes zoster reactivation following intra-articular corticosteroid injection is uncommon, and cutaneous small-vessel vasculitis represents a rare manifestation of VZV infection. Although each has been described independently, to our knowledge, their coexistence following intra-articular corticosteroid administration in an immunocompetent patient has not been previously reported.

A potential explanation for herpes zoster reactivation in our patient is the immunomodulatory effect of corticosteroids. Despite their localized administration, intra-articular corticosteroid injections can produce systemic effects,^8^ including transient suppression of T-cell-mediated immunity and cytokine signaling, thereby facilitating reactivation of latent VZV, even in otherwise immunocompetent individuals. The absolute risk of herpes zoster, however, remains low among otherwise healthy individuals receiving corticosteroids. For example, a large population-based cohort study demonstrated an incidence of approximately 15 cases per 1000 person-years among healthy patients receiving systemic corticosteroids.^3^ Consistent with this, reports of herpes zoster following intra-articular corticosteroid injection are exceedingly limited. To our knowledge, only 2 such cases have been described in the literature: 1 case of localized herpes zoster following intra-articular knee injection in an 80-year-old man with multiple comorbidities, and 1 case of Ramsay Hunt syndrome after bilateral intra-articular knee injections in a 55-year-old woman without significant comorbidities.^4^^,^^5^

Our findings also suggest that localized trauma may represent an additional contributing factor. Consistent with this, a large case–control study of over 80,000 Medicare beneficiaries demonstrated that recent physical trauma was associated with 3.4-fold higher odds of herpes zoster, with even stronger associations observed following cranial trauma (25-fold higher odds).^9^ Local trauma may play a role in viral reactivation through stimulation of sensory nerves with subsequent reactivation within the corresponding dorsal root ganglion, coupled with localized immune dysregulation.

In addition to trauma-related triggers, host immune status may influence the severity and clinical manifestations of VZV infection. In 1 case series of 5 patients with VZV-associated segmental leukocytoclastic vasculitis, four were immunocompromised (including patients with sarcoidosis, mycosis fungoides, and glomerular disease), and notably, 4 cases demonstrated involvement of the lower extremities, similar to our patient.^10^ However, 1 patient in this series was immunocompetent with involvement of the lower extremity, highlighting that such presentations may also occur in the absence of systemic immunosuppression, consistent with our findings. Proposed mechanisms of VZV-associated vasculitis include immune complex–mediated vascular injury, dysregulated immune responses with autoreactive cell activation, and direct viral involvement of the endothelium leading to vascular remodeling.^10^

This case expands the clinical spectrum of VZV reactivation and suggests that even localized corticosteroid administration may be sufficient to precipitate both viral reactivation and secondary vasculitic manifestations in immunocompetent individuals. In patients presenting with unusual cutaneous findings following intra-articular corticosteroid injection, consideration of VZV infection is warranted, and histopathologic evaluation with viral testing can help establish the diagnosis. Prompt recognition is critical, as early initiation of antiviral therapy may improve outcomes and prevent disease progression. Although topical corticosteroids do not have an established role in the routine management of herpes zoster, they may be considered for symptomatic relief in select cases with associated inflammatory vasculitic changes, with careful clinical judgment.

Given the widespread use of intra-articular corticosteroid injections, awareness of this rare complication is important to avoid misdiagnosis and delayed antiviral therapy.

## Conflicts of interest

None disclosed.
